# What can neuroimmunology teach us about the symptoms of long-COVID?

**DOI:** 10.1093/oxfimm/iqab004

**Published:** 2021-02-10

**Authors:** Valeria Mondelli, Carmine M Pariante

**Affiliations:** 1 Department of Psychological Medicine, Institute of Psychiatry, Psychology and Neuroscience, King’s College London, London, UK; 2 National Institute for Health Research Mental Health Biomedical Research Centre, South London and Maudsley NHS Foundation Trust and King's College London, London, UK

**Keywords:** COVID-19, long-COVID, fatigue, microglia, depression, neuroimmunology

## Abstract

Long-Coronavirus Disease (Long-COVID) is becoming increasingly recognized due to the persistence of symptoms such as profound fatigue, neurocognitive difficulties, muscle pains and weaknesses and depression, which would last beyond 3–12 weeks following infection with SARS-CoV-2. These particular symptoms have been extensively observed and studied in the context of previous psychoneuroimmunology research. In this short commentary, we discuss how previous neuroimmunology studies could help us to better understand pathways behind the development of these prolonged symptoms. Various mechanisms, including viral neuroinvasion, glial cells activation, neurogenesis, oxidative stress have been shown to explain these symptoms in the context of other disorders. Previous neuroimmunology findings could represent helpful pointers for future research on long-COVID symptoms and suggest potential management strategies for patients suffering with long-COVID.

## INTRODUCTION

COVID-19 has been initially believed to be a relatively short-term illness, lasting between 1 and 3 weeks. However, it is becoming increasingly acknowledged that a number ranging from 10% to over 80% of patients infected with SARS-CoV-2 experience symptoms beyond 3 weeks and sometimes beyond 12 weeks [1, 2]. The percentage of patients presenting with prolonged symptoms varies according to the studies which focussed on different durations of follow-up and types of population (e.g. patients in the community vs. those attending hospital/specialist clinics); the rate of patients with prolonged symptoms was reported between 10%, when considering patients from a community sample who remained unwell beyond 3 weeks [1], and 87%, when considering those who were initially hospitalized and reported at least one persistent symptom beyond the first month [3]. Symptoms of long-COVID can vary widely and include cough, low-grade fever, fatigue, chest pain, shortness of breath, headaches, neurocognitive difficulties, muscle pain and weaknesses, gastrointestinal upset, rashes, metabolic disruption, thromboembolic conditions, and depression and other mental health conditions [1, 4].

The persistence of some of these symptoms is not particularly surprising in the context of what we have learned over the past two decades from studies in the field of neuroimmunology [5–8]. In recent years, we have witnessed a surge in number of studies reporting immune abnormalities, showing predominantly an increase in the activity of the innate immune system, in conditions such as chronic fatigue syndrome/myalgic encephalomyelitis [6, 8], fibromyalgia [9], cognitive dysfunction [10], depression and other mental health disorders [7, 11, 12]. Here we discuss evidence that support the potential involvement of neuroimmunology pathways in the development of prolonged COVID-19 symptoms and implications for future research and management strategies.

## POTENTIAL EFFECTS OF SARS-CoV-2 ON CENTRAL NERVOUS SYSTEM

A number of causes have been so far suggested to potentially explain prolonged COVID-19 symptoms, including the presence of persistent low viral load, reinfection, changes in immune cells activity potentially leading to autoimmune responses and tissue damage caused by the initial viral infection [1]. The damage of the central nervous system (CNS) and the involvement of neuroimmunological pathways could be particularly relevant for symptoms such as persistent fatigue, cognitive dysfunction, headaches, muscle weaknesses, depression and other mental health symptoms. Evidence supporting an involvement of CNS in SARS-CoV-2 infection comes from studies showing neurological, psychiatric and neuropsychiatric presentations in the context of confirmed COVID-19 cases [4, 13, 14].

How a virus, which was originally thought only to affect respiratory system, could affect the CNS has been the focus of various studies and commentaries in the past few months. Various viral infections are known to potentially cause important damage to structure and function of CNS and lead to onset of encephalitis, toxic encephalopathies or demyelinating lesions [15]. This has been previously partially explained by the ability of these viruses to ‘invade’ the CNS, potentially damaging the blood–brain barrier [16], causing direct nerve damage, and activating microglia and astrocytes in the brain and leading to a pro-inflammatory state at central level [17]. SARS-CoV-2 RNA transcripts have been detected in a small number of human brain tissues from recent autopsy studies [18, 19], supporting the idea that this virus could affect CNS and brain tissue. Furthermore, a very recent study [20] has provided evidence for neuroinvasive capacity of SARS-CoV-2 and identified pathological features with minimal immune cells infiltrates. Very few neuropathological post-mortem studies of patients with COVID-19 have been published so far; among these, the most recent comprehensive report shows evidence of ischemic lesions in ∼14% of the patients, a variable degree of astrogliosis across different brain regions in the majority (86%) of the patients examined, and evidence of activation of microglia and infiltration of cytotoxic T lymphocytes mainly in the brainstem and cerebellum [21]. However, some studies have not been able to detect viral invasion even though showing evidence of inflammation in the cerebrospinal fluid [22, 23]. Interestingly, the presence of SARS-CoV-2 in the CNS has not been associated directly with the severity of the neuropathological findings, suggesting that neuroinvasion may be only one of the pathways through which SARS-CoV-2 could influence brain function and contribute to some of the prolonged COVID-19 symptoms.

Peripheral inflammation has been widely recognized to influence brain function through activation of glial cells such as microglia and astrocytes, by influencing neurogenesis, metabolism of neurotransmitters and stress response [24–27]. Studies using peripheral immune challenges (such as interferon (IFN)-alpha, lipopolysaccharide, typhoid vaccine) for therapeutic and experimental purposes have clearly documented onset of symptoms such as fatigue and depression and effects on brain function and activity [8, 28–31] although a dose–response effects between severity of inflammation and intensity of the symptoms are not always evident, especially if the symptoms starts weeks after the initial immune challenge. The lack of clear dose–response effect could be due to various factors that could moderate the effects of inflammation on the brain; these could, e.g. include changes in permeability of the blood–brain barrier or the presence of glucocorticoid resistance, both important for regulating potential effects of increased immune activation on the brain. Indeed, if there was a clear dose–response, all patients with inflammatory disorders would present with mental health or neurological problems; in contrast, it is evident from previous studies that even a relatively low level of immune activation, similar to that reported in individuals with cardiovascular risk, can be associated with onset of psychiatric symptoms/disorders. Below we will summarize some of the relevant evidence from neuroimmunology studies focussing on specific symptoms relevant to long-COVID.

## WHAT HAVE WE LEARNT FROM NEUROIMMUNOLOGY THAT IS RELEVANT FOR LONG-COVID SYMPTOMS?

The last two decades have seen a large number of studies showing increased levels of peripheral inflammation in patients with mental health disorders and a specific association with some of the symptoms relevant to long-COVID. Chronic fatigue syndrome has been previously described following infections with other coronaviruses (SARS and MERS) [32, 33] and other viruses, such as Epstein–Barr virus [34]. Previous studies have reported increased inflammatory markers such as C reactive protein, white blood cell count and transforming growth factor (TGF)-beta, in patients with chronic fatigue syndrome [6, 35]. Elevated levels of TGF-beta in patients with chronic fatigue syndrome were also one of the main findings in a more recent study, which also reported an association between severity of symptoms and the levels of a wide range of pro-inflammatory cytokines, including CCL11, CXCL10, IFN-gamma and interleukin (IL)-5, but not a directly association between symptoms severity and TGF-beta levels [36]. However, increased levels of inflammatory markers are not always detected in individuals with chronic fatigue syndrome, as reported by a recent meta-analysis which highlights that other mechanisms may be more relevant for the etiopathogenesis of this disorder [37]. Nevertheless, central inflammation and cytokine secretion appear to be essential steps for the development of fatigue in the context of psychiatric and non-psychiatric disorders. Recent preclinical evidence has shown that microglia could have a key role in the development of fatigue, where induction of fatigue appeared to be dependent on the production of IL-1 beta from activated microglia [38, 39]. Similar to chronic fatigue syndrome, patients with fibromyalgia, a condition characterized by widespread pain, cognitive impairment, sleep disturbances and persistent lack of energy, also present with increased levels of inflammatory markers [40]. Microglia, and in particular hypersensitive microglia, has been also recently suggested to play a role in the development of fibromyalgia [41]. Over-activation of microglial cells is a common feature also in patients with chronic pain [42]; of note, an over-reaction of glial cells exposed to SARS-CoV-2 has been also recently suggested as one of the main mechanisms involved in the neuropsychiatric symptoms of COVID-19 [43]. Indeed, an important conundrum in these studies is that a direct correlation between the magnitude of the peripheral immune activation and the severity of fatigue and pain is not immediately evident [44]. This is indeed relevant also for long-COVID as persistent fatigue following SARS-CoV-2 has not been found to be dependent on the severity of the initial infection or associated with levels of peripheral inflammatory markers [45]. A number of non-mutually exclusive explanations have been proposed, such as that serum immune markers might be unable to capture the complexity of relevant immune mechanisms; that the individual sensitivity to immune activation might be more important than the absolute levels of immune activation per se; or that central processes involving brain/microglia response may be more relevant and persist even when the initial trigger from peripheral immune activation has resolved and therefore no longer measurable [12]. In this context, the blood–brain barrier may represent a potential important factor moderating the crosstalk between peripheral immune and CNS. We know that pro-inflammatory cytokines can directly increase blood–brain barrier permeability partly by upregulating endothelial cell adhesion molecules and promoting immune cells infiltration [46]. However, peripheral inflammation does not always lead to increased peripheral permeability of the blood–brain barrier, and it has been associated in certain cases with reduced permeability [47]. An increased/reduced permeability of the blood–brain barrier could partly be behind the dissociation between the levels of peripheral inflammation and the severity of symptoms such as fatigue, depression or cognitive impairment.

Depression and other mental health problems have also been increasingly associated with chronic low-grade inflammation and microglia activation [7, 11, 12]. Interestingly, cognitive dysfunction appears to be one of the main symptoms linked to increased levels of inflammatory markers [10, 48]. The molecular pathways through which central inflammation contributes to depression and cognitive dysfunction are still partly unclear, but preclinical evidence suggests that central neuroinflammation may contribute to reduced neurogenesis particularly in the hippocampus, one of the main structures involved in cognitive function and memory [26, 48]. Other potential pathways include effects of synthesis of serotonin following the activation of kynurenine pathways [49, 50] and an unbalance in oxidative stress markers [39]. More specifically, cytokines can activate a key enzyme in the kynurenine pathway, the indolamine 2,3-dioxygenase (IDO); IDO catabolizes the amino acid tryptophan, essential precursor of the neurotransmitter serotonin into different metabolic products, namely the quinolinic and kynurenic acid [51]. Quinolinic acid is regarded as neurotoxic while kynurenic acid is neuroprotective and both metabolites interact with glutamate neurotransmitter system. The activation of the kynurenine pathway has been suggested to play a role in the development of depression also in the context of other physical health conditions associated with important activation of the immune system [50]. Similarly, increased oxidative stress has been associated with development of depression and with neurodegenerative disorders [52–54]. Oxidative stress derives from an imbalance between reactive oxygen species and antioxidants; increased oxidative stress can result in production of toxic by-products which would affect cell growth, proliferation and differentiation and lead ultimately to neuronal cell death. Immune cell activation and inflammation can contribute to the production of endogenous free radicals and lead to an increase of oxidative stress; on the other hand, oxidants are known to enhance inflammation via activation of transcription factors such as NF-kappa B. This positive feedback loop has been suggested to contribute to neuronal damage and persistence of symptoms of depression and cognitive impairment [53].

## USING OUR KNOWLEDGE FROM NEUROIMMUNOLOGY TO IMPROVE OUR UNDERSTANDING AND MANAGEMENT OF LONG-COVID

Most of us who have worked in the field of psychoneuroimmunology for years are not surprised by the high rate of patients with persistent fatigue, cognitive dysfunction, muscle pains, depression and other mental health problems in patients who have been infected by SARS-CoV-2. These symptoms have been long acknowledged in the context of different disorders and syndromes to have a strong link with an initial immune challenge and/or with a persisting dysregulation of the immune system. We have learned over the years that even mild infections and low-grade inflammation, as the one reported in cardiovascular illnesses, can cause symptoms of depression or be associated with persistent fatigue. In this short commentary, we have discussed few possible pathways that could be relevant for some of these symptoms, including the involvement of activation of glial cells, blood–brain barrier permeability, effects of oxidative stress and kynurenine pathway. These are just few pointers for future research on the causes of long-COVID symptoms and for a better understanding of management and support strategies.

Treatment strategies currently trialled for patients with depression presenting with increased inflammation include the use of anti-inflammatory medications, such as minocycline and tumor necrosis factor (TNF)-alpha inhibitors [55]. However, we need to remember that the communication between CNS and immune system is bidirectional [56]; therefore, we could potentially reduce persistent low-grade inflammation by modulating the immune system through a neural/endocrine pathway. Indeed, psychosocial factors are also very important in regulating our immune activation and the response to SARS-CoV-2 [57]. Two of the main biological systems involved in the stress response, the hypothalamic–pituitary–adrenal axis or the autonomic nervous system, are also key in the regulation of our immune response. Therefore, strategies tackling our levels of stress and/or the stress response, including psychosocial intervention, physical exercise or potentially dietary interventions, could be also useful in counteracting some of the negative effects of chronic inflammation.

In conclusion, the neuroimmunology knowledge acquired over the years about chronic fatigue syndrome, fibromyalgia, depression and mental health disorders could potentially assist research and understanding of many long-COVID symptoms.

## References

[1] Greenhalgh T , Knight M, A'Court C, Buxton M et al Management of post-acute covid-19 in primary care. BMJ 2020;370:m3026. 32784198 10.1136/bmj.m3026

[2] Study CS. How Long Does COVID-19 Last? https://covid.joinzoe.com/post/covid-long-term?fbclid=IwAR1RxIcmmdL-EFjh_aI- (6 June 2020, date last accessed).

[3] Carfi A , Bernabei R, Landi F, Gemelli Against C-P-ACSG. Persistent symptoms in patients after acute COVID-19. JAMA 2020;324:603–5. 32644129 10.1001/jama.2020.12603 PMC7349096

[4] Mazza MG , De Lorenzo R, Conte C et al Anxiety and depression in COVID-19 survivors: role of inflammatory and clinical predictors. Brain Behav Immun 2020;89:594–600. 32738287 10.1016/j.bbi.2020.07.037 PMC7390748

[5] Dantzer R , O'Connor JC, Freund GG et al From inflammation to sickness and depression: when the immune system subjugates the brain. Nat Rev Neurosci 2008;9:46–56. 18073775 10.1038/nrn2297 PMC2919277

[6] Raison CL , Lin JM, Reeves WC. Association of peripheral inflammatory markers with chronic fatigue in a population-based sample. Brain Behav Immun 2009;23:327–37. 19111923 10.1016/j.bbi.2008.11.005

[7] Mondelli V , Vernon AC, Turkheimer F et al Brain microglia in psychiatric disorders. Lancet Psychiatry 2017;4:563–72. 28454915 10.1016/S2215-0366(17)30101-3

[8] Russell A , Hepgul N, Nikkheslat N et al Persistent fatigue induced by interferon-alpha: a novel, inflammation-based, proxy model of chronic fatigue syndrome. Psychoneuroendocrinology 2019;100:276–85. 30567628 10.1016/j.psyneuen.2018.11.032 PMC6350004

[9] Albrecht DS , Forsberg A, Sandstrom A et al Brain glial activation in fibromyalgia - a multi-site positron emission tomography investigation. Brain Behav Immun 2019;75:72–83. 30223011 10.1016/j.bbi.2018.09.018 PMC6541932

[10] Marsland AL , Gianaros PJ, Kuan DC et al Brain morphology links systemic inflammation to cognitive function in midlife adults. Brain Behav Immun 2015;48:195–204. 25882911 10.1016/j.bbi.2015.03.015 PMC4508197

[11] Baumeister D , Russell A, Pariante CM et al Inflammatory biomarker profiles of mental disorders and their relation to clinical, social and lifestyle factors. Soc Psychiatry Psychiatr Epidemiol 2014;49:841–9. 24789456 10.1007/s00127-014-0887-z

[12] Enache D , Pariante CM, Mondelli V. Markers of central inflammation in major depressive disorder: a systematic review and meta-analysis of studies examining cerebrospinal fluid, positron emission tomography and post-mortem brain tissue. Brain Behav Immun 2019;81:24–40. 31195092 10.1016/j.bbi.2019.06.015

[13] Paterson RW , Brown RL, Benjamin L et al The emerging spectrum of COVID-19 neurology: clinical, radiological and laboratory findings. Brain 2020;143:3104–20. 32637987 10.1093/brain/awaa240 PMC7454352

[14] Rogers JP , Chesney E, Oliver D et al Psychiatric and neuropsychiatric presentations associated with severe coronavirus infections: a systematic review and meta-analysis with comparison to the COVID-19 pandemic. Lancet Psychiatry 2020;7:611–27. 32437679 10.1016/S2215-0366(20)30203-0 PMC7234781

[15] Wu Y , Xu X, Chen Z et al Nervous system involvement after infection with COVID-19 and other coronaviruses. Brain Behav Immun 2020;87:18–22. 32240762 10.1016/j.bbi.2020.03.031 PMC7146689

[16] Michalicova A , Bhide K, Bhide M et al How viruses infiltrate the central nervous system. Acta Virol 2017;61:393–400. 29186956 10.4149/av_2017_401

[17] Li Y , Fu L, Gonzales DM et al Coronavirus neurovirulence correlates with the ability of the virus to induce proinflammatory cytokine signals from astrocytes and microglia. J Virol 2004;78:3398–406. 15016862 10.1128/JVI.78.7.3398-3406.2004 PMC371061

[18] Puelles VG , Lutgehetmann M, Lindenmeyer MT et al Multiorgan and renal tropism of SARS-CoV-2. N Engl J Med 2020;383:590–2. 32402155 10.1056/NEJMc2011400 PMC7240771

[19] Solomon IH , Normandin E, Bhattacharyya S et al Neuropathological features of Covid-19. N Engl J Med 2020;383:989–92. 32530583 10.1056/NEJMc2019373 PMC7304421

[20] Song E , Zhang C, Israelow B et al Neuroinvasion of SARS-CoV-2 in human and mouse brain. bioRxiv 2020. 10.1084/jem.20202135 PMC7808299 33433624

[21] Matschke J , Lutgehetmann M, Hagel C et al Neuropathology of patients with COVID-19 in Germany: a post-mortem case series. Lancet Neurol 2020;19:919–29. 33031735 10.1016/S1474-4422(20)30308-2 PMC7535629

[22] Bernard-Valnet R , Pizzarotti B, Anichini A et al Two patients with acute meningoencephalitis concomitant with SARS-CoV-2 infection. Eur J Neurol 2020;27:e43–4. 32383343 10.1111/ene.14298 PMC7267660

[23] Ye M , Ren Y, Lv T. Encephalitis as a clinical manifestation of COVID-19. Brain Behav Immun 2020;88:945–6. 32283294 10.1016/j.bbi.2020.04.017 PMC7146652

[24] Felger JC, , Li L, Marvar PJ et al Tyrosine metabolism during interferon-alpha administration: association with fatigue and CSF dopamine concentrations. Brain Behav Immun 2013;31:153–60. 23072726 10.1016/j.bbi.2012.10.010 PMC3578984

[25] Borsini A , Cattaneo A, Malpighi C et al Interferon-alpha reduces human hippocampal neurogenesis and increases apoptosis via activation of distinct STAT1-dependent mechanisms. Int J Neuropsychopharmacol 2018;21:187–200. 29040650 10.1093/ijnp/pyx083 PMC5793815

[26] Borsini A , Di Benedetto MG, Giacobbe J et al Pro- and anti-inflammatory properties of interleukin (IL6) in vitro: relevance for major depression and for human hippocampal neurogenesis. Int J Neuropsychopharmacol 2020;23;738–50. 10.1093/ijnp/pyaa055 PMC7745251 32726406

[27] Zheng LS , Kaneko N, Sawamoto K. Minocycline treatment ameliorates interferon-alpha-induced neurogenic defects and depression-like behaviors in mice. Front Cell Neurosci 2015;9:5. 25674053 10.3389/fncel.2015.00005 PMC4309184

[28] Sandiego CM , Gallezot JD, Pittman B et al Imaging robust microglial activation after lipopolysaccharide administration in humans with PET. Proc Natl Acad Sci USA 2015;112:12468–73. 26385967 10.1073/pnas.1511003112 PMC4603509

[29] Nettis MA , Veronese M, Nikkheslat N et al PET imaging shows no changes in TSPO brain density after IFN-alpha immune challenge in healthy human volunteers. Trans Psychiatry 2020;10:89. 10.1038/s41398-020-0768-z PMC7063038 32152285

[30] Dowell NG , Bouyagoub S, Tibble J et al Interferon-alpha-induced changes in NODDI predispose to the development of fatigue. Neuroscience 2019;403:111–7. 29292074 10.1016/j.neuroscience.2017.12.040 PMC6458994

[31] Harrison NA , Brydon L, Walker C et al Inflammation causes mood changes through alterations in subgenual cingulate activity and mesolimbic connectivity. Biol Psychiatry 2009;66:407–14. 19423079 10.1016/j.biopsych.2009.03.015 PMC2885494

[32] Lam MH , Wing YK, Yu MW et al Mental morbidities and chronic fatigue in severe acute respiratory syndrome survivors: long-term follow-up. Arch Intern Med 2009;169:2142–7. 20008700 10.1001/archinternmed.2009.384

[33] Moldofsky H , Patcai J. Chronic widespread musculoskeletal pain, fatigue, depression and disordered sleep in chronic post-SARS syndrome; a case-controlled study. BMC Neurol 2011;11:37. 21435231 10.1186/1471-2377-11-37 PMC3071317

[34] Straus SE , Tosato G, Armstrong G et al Persisting illness and fatigue in adults with evidence of Epstein-Barr virus infection. Ann Intern Med 1985;102:7–16. 2578268 10.7326/0003-4819-102-1-7

[35] Blundell S , Ray KK, Buckland M et al Chronic fatigue syndrome and circulating cytokines: a systematic review. Brain Behav Immun 2015;50:186–95. 26148446 10.1016/j.bbi.2015.07.004

[36] Montoya JG , Holmes TH, Anderson JN et al Cytokine signature associated with disease severity in chronic fatigue syndrome patients. Proc Natl Acad Sci USA 2017;114:E7150–8. 28760971 10.1073/pnas.1710519114 PMC5576836

[37] Strawbridge R , Sartor ML, Scott F et al Inflammatory proteins are altered in chronic fatigue syndrome-a systematic review and meta-analysis. Neurosci Biobehav Rev 2019;107:69–83. 31465778 10.1016/j.neubiorev.2019.08.011

[38] Ifuku M , Hossain SM, Noda M et al Induction of interleukin-1beta by activated microglia is a prerequisite for immunologically induced fatigue. Eur J Neurosci 2014;40:3253–63. 25040499 10.1111/ejn.12668

[39] Chaves-Filho AJM , Macedo DS, de Lucena DF et al Shared microglial mechanisms underpinning depression and chronic fatigue syndrome and their comorbidities. Behav Brain Res 2019;372:111975. 31136774 10.1016/j.bbr.2019.111975

[40] Romano GF , Tomassi S, Russell A et al Fibromyalgia and chronic fatigue: the underlying biology and related theoretical issues. Adv Psychosom Med 2015;34:61–77. 25832514 10.1159/000369085

[41] Ohgidani M , Kato TA, Hosoi M et al Fibromyalgia and microglial TNF-alpha: translational research using human blood induced microglia-like cells. Sci Rep 2017;7:11882. 28928366 10.1038/s41598-017-11506-4 PMC5605512

[42] Donnelly CR , Andriessen AS, Chen G et al Central nervous system targets: glial cell mechanisms in chronic pain. Neurotherapeutics 2020;17:846–60. 32820378 10.1007/s13311-020-00905-7 PMC7609632

[43] Tremblay ME , Madore C, Bordeleau M et al Neuropathobiology of COVID-19: the role for glia. Front Cell Neurosci 2020;14:592214. 33304243 10.3389/fncel.2020.592214 PMC7693550

[44] Menzies V , Lyon DE, Elswick RK Jr et al Psychoneuroimmuno-logical relationships in women with fibromyalgia. Biol Res Nurs 2013;15:219–25. 22174319 10.1177/1099800411424204 PMC3722552

[45] Townsend L , Dyer AH, Jones K et al Persistent fatigue following SARS-CoV-2 infection is common and independent of severity of initial infection. PLoS One 2020;15:e0240784. 33166287 10.1371/journal.pone.0240784 PMC7652254

[46] Hayley S , Hakim AM, Albert PR. Depression, dementia and immune dysregulation. Brain 2020. 10.1093/brain/awaa405 PMC8041341 33279966

[47] Turkheimer FE , Althubaity N, Schubert J et al Increased serum peripheral C-reactive protein is associated with reduced brain barriers permeability of TSPO radioligands in healthy volunteers and depressed patients: implications for inflammation and depression. Brain Behav Immun 2020;91:487–97. 33160089 10.1016/j.bbi.2020.10.025

[48] Yirmiya R , Goshen I. Immune modulation of learning, memory, neural plasticity and neurogenesis. Brain Behav Immun 2011;25:181–213. 20970492 10.1016/j.bbi.2010.10.015

[49] Dantzer R. Role of the kynurenine metabolism pathway in inflammation-induced depression: preclinical approaches. Curr Top Behav Neurosci 2017;31:117–38. 27225497 10.1007/7854_2016_6 PMC6585430

[50] Sforzini L , Nettis MA, Mondelli V et al Inflammation in cancer and depression: a starring role for the kynurenine pathway. Psychopharmacology (Berl) 2019;236:2997–3011. 30806743 10.1007/s00213-019-05200-8 PMC6820591

[51] Dantzer R , O'Connor JC, Lawson MA et al Inflammation-associated depression: from serotonin to kynurenine. Psychoneuroendocrinology 2011;36:426–36. 21041030 10.1016/j.psyneuen.2010.09.012 PMC3053088

[52] Hepgul N , Cattaneo A, Agarwal K et al Transcriptomics in interferon-alpha-treated patients identifies inflammation-, neuroplasticity- and oxidative stress-related signatures as predictors and correlates of depression. Neuropsychopharmacology 2016;41:2502–11. 27067128 10.1038/npp.2016.50 PMC4983179

[53] Bakunina N , Pariante CM, Zunszain PA. Immune mechanisms linked to depression via oxidative stress and neuroprogression. Immunology 2015;144:365–73. 25580634 10.1111/imm.12443 PMC4557673

[54] Arslan J , Jamshed H, Qureshi H. Early detection and prevention of Alzheimer's disease: role of oxidative markers and natural antioxidants. Front Aging Neurosci 2020;12:231. 32848710 10.3389/fnagi.2020.00231 PMC7397955

[55] Raison CL , Rutherford RE, Woolwine BJ et al A randomized controlled trial of the tumor necrosis factor antagonist infliximab for treatment-resistant depression: the role of baseline inflammatory biomarkers. JAMA Psychiatry 2013;70:31–41. 22945416 10.1001/2013.jamapsychiatry.4 PMC4015348

[56] Pariante CM. Psychoneuroimmunology or immunopsychiatry? Lancet Psychiatry 2015;2:197–9. 26359887 10.1016/S2215-0366(15)00042-5 PMC4580988

[57] Batty GD , Deary IJ, Luciano M et al Psychosocial factors and hospitalisations for COVID-19: prospective cohort study based on a community sample. Brain Behav Immun 2020. 10.1016/j.bbi.2020.06.021 PMC7297693 32561221

